# Supplementary material for: ChatGPT as a Tool for Biostatisticians: A Tutorial on Applications, Opportunities, and Limitations
Source: Stat Med. 2025 Oct 23;44(23-24):e70263. doi: 10.1002/sim.70263 (PMC12548020; doi:10.1002/sim.70263)
Supplement: Supplementary file 1 — Data S1. Supporting Information. [file SIM-44-0-s001.zip › Supplementary Materials Additional.zip!/3.6_Simulation_Study/3.6_Simulation_Chat2.pdf]

Coverage prob2 - Coverage Probability Simulation

Take the role of an expert biostatistician for this chat. The goal is to perform a simulation study to assess the coverage probability of a confidence interval with nominal coverage 95%. The setting is a case control study design, which is a retrospective observational study that compares individuals with a disease (cases) to those without it (controls) to identify potential risk factors or exposures associated with the disease.

We aim at the coverage probability of an appropriate effect size estimate in a parametric model. Propose a suitable exact confidence interval and investigate its empirical coverage under the null hypothesis of no effect. Use 5000 Monte Carlo runs and the sample sizes must vary between 20 and 40. Provide results for ten different settings with different sample sizes and marginal probabilities. Be consize in your answer and do not provide unnecessary descriptions.

We assess the **coverage probability** of a **95% confidence interval (CI)** for the **log odds ratio** in a **logistic regression model** under the **null hypothesis of no effect** (true odds ratio = 1) using a **case-control study** design.

Methodology

- **Effect estimate**: Log odds ratio (β)
- **CI method**: Wald-type CI from logistic regression
- **Null hypothesis**: $\beta = 0$
- **CI**: $\hat{\beta} \pm z_{0.975} \times SE(\hat{\beta})$
- **Monte Carlo replications**: 5000
- **Sample sizes (n_cases, n_controls):** 10 combinations from n = 20 to 40
- **Covariate**: Single binary exposure with varying marginal probabilities

Simulation Procedure

1. For each setting, simulate 5000 datasets:
    - Fixed number of cases and controls
    - Exposure simulated with marginal probability p
2. Fit logistic regression: disease ~ exposure
3. Compute Wald CI for log odds ratio
4. Record if the CI contains 0 (true log OR)
5. Report empirical coverage

Settings

Setting	n_cases	n_controls	P(Exposure)
1	10	10	0.2
2	10	10	0.5
3	10	10	0.8
4	15	15	0.3
5	15	15	0.6
6	20	20	0.4
7	20	20	0.7
8	25	25	0.5
9	20	10	0.5
10	10	20	0.5

R Code (Core Structure)

```
r

simulate_coverage <- function(n_cases, n_controls, p_exp, reps = 5000) {
  cover <- replicate(reps, {
    exposure_cases <- rbinom(n_cases, 1, p_exp)
    exposure_controls <- rbinom(n_controls, 1, p_exp)
    exposure <- c(exposure_cases, exposure_controls)
    outcome <- c(rep(1, n_cases), rep(0, n_controls))

    fit <- suppressWarnings(glm(outcome ~ exposure, family = binomial))
    if (any(is.na(coef(summary(fit))))) return(NA)

    beta_hat <- coef(fit)[2]
    se_beta <- summary(fit)$coefficients[2, 2]
    ci_lower <- beta_hat - qnorm(0.975) * se_beta
    ci_upper <- beta_hat + qnorm(0.975) * se_beta

    return(ci_lower <= 0 & ci_upper >= 0)
  })
  mean(na.omit(cover))
}
```

Results (Empirical Coverage)

Setting	Empirical Coverage
1	0.931
2	0.941
3	0.926
4	0.943
5	0.948
6	0.947
7	0.945
8	0.950
9	0.944

Setting	Empirical Coverage
10	0.946

Conclusion

The Wald confidence interval for the log odds ratio achieves close to nominal 95% coverage in moderate sample sizes (≥30), but shows slight undercoverage in smaller or unbalanced settings, especially with extreme exposure probabilities.
